# Early Implementation of QbD in Biopharmaceutical Development: A Practical Example

**DOI:** 10.1155/2015/605427

**Published:** 2015-05-17

**Authors:** Jesús Zurdo, Andreas Arnell, Olga Obrezanova, Noel Smith, Ramón Gómez de la Cuesta, Thomas R. A. Gallagher, Rebecca Michael, Yvette Stallwood, Caroline Ekblad, Lars Abrahmsén, Ingmarie Höidén-Guthenberg

**Affiliations:** ^1^Research and Technology, Pharma & Biotech, Lonza Biologics PLC, Granta Park, Great Abington, Cambridge CB21 6GS, UK; ^2^Applied Protein Services, Lonza Biologics PLC, Granta Park, Great Abington, Cambridge CB21 6GS, UK; ^3^Affibody AB, Gunnar Asplunds Allé 24, 171 63 Solna, Sweden

## Abstract

In drug development, the “onus” of the low R&D efficiency has been put traditionally onto the drug discovery process (i.e., finding the right target or “binding” functionality). Here, we show that manufacturing is not only a central component of product success, but also that, by integrating manufacturing and discovery activities in a “holistic” interpretation of QbD methodologies, we could expect to increase the efficiency of the drug discovery process as a whole. In this new context, early risk assessment, using developability methodologies and computational methods in particular, can assist in reducing risks during development in a cost-effective way. We define specific areas of risk and how they can impact product quality in a broad sense, including essential aspects such as product efficacy and patient safety. Emerging industry practices around developability are introduced, including some specific examples of applications to biotherapeutics. Furthermore, we suggest some potential workflows to illustrate how developability strategies can be introduced in practical terms during early drug development in order to mitigate risks, reduce drug attrition and ultimately increase the robustness of the biopharmaceutical supply chain. Finally, we also discuss how the implementation of such methodologies could accelerate the access of new therapeutic treatments to patients in the clinic.

## 1. Introduction

Failure of new therapeutic candidates during development is unfortunately a very common occurrence. Recent estimates show that, on average, pharmaceutical companies seem to spend between four and eleven billion US dollars for every new therapeutic treatment that is eventually commercialised (Forbes—the truly staggering cost of inventing new drugs. http://goo.gl/C2KSB). The main reason for this is fundamentally the extraordinarily high rate of failure observed during drug development. Approximately 90% of drug candidates will fail during clinical development; maybe over 99% if preclinical stages of development are also included (PhRMA. http://phrma.org/) [1]. This level of failure is further compounded with increasing expectations from payers in terms of therapeutic outcomes and value for money. As a result, there is a growing interest in maximising the return on the investment made in the development of new therapeutic candidates, avoiding whenever possible late and expensive failures. However, the true reasons behind drug failure during development remain a highly debated and poorly understood issue for many, primarily due to the lack of detailed and up-to-date data on the subject. This occurs either because of lack of public data on the reasons behind development discontinuation, or because a combination of different elements often play a role in the demise of a particular drug candidate, making it difficult to identify specific contributing factors. Kola and Landis [2] and other analyses published since [3] have shed some light on the subject, suggesting a collection of different causes behind drug attrition.

Inadequate efficacy is perhaps “the” major single reason behind clinical failure, but other relevant causes include bioavailability and pharmacology shortcomings, safety and toxicology problems, or even stability and quality issues. Furthermore, strategic and commercial plans are scrutinised ever more closely as health care providers in many countries demand more value for their money. Discontinuation can, therefore, not only hint to problems with the design but also cost of goods, insufficient demand at the required pricing/available reimbursement, lack of competitive advantage over other products in the market or under development, and even insufficient available investment to complete development. Whereas the failure of new drug candidates during late clinical development and registration is primarily linked to inadequate biological activity and efficacy or pharmacology and dosage issues [4], attrition during early clinical development fundamentally relates to problems with safety (immunogenic reactions or hypersensitivity in biopharmaceuticals) and, less often, pharmacology. Preclinical drug attrition is a very complex area to survey, but manufacturing and quality issues related to product stability and even productivity are common problems observed.

The tragic consequence of the current fragmented approach to drug development is that key design elements that are essential for the success of new therapeutics can inadvertently be left out during discovery and early development stages of new drug candidates. Whilst strides have indeed been made in recent years to address at least some of these risk aspects early on in development, the methodologies employed are still far from being robust and efficient and many gaps do still exist [5]. Such gaps can often cause significant problems that are only discovered quite late in development. Severe delays can ensue, requiring additional investments or, in some cases, trigger the discontinuation of an entire drug development programme. Indeed, delays in development, reworkings, failed batches, or deviations are all frequent and costly issues observed during development and manufacturing of biological products and, in many occasions, can ultimately be traced back to poor design of the product candidate and/or manufacturing process.

From a biopharmaceutical development perspective, a significant financial commitment is made for the development of a qualified manufacturing process well before the product has even been cleared for its assessment in clinical trials. In fact, almost fully commercially defined processes are usually developed for “prototypes” (drug candidates) that, in a majority of cases, will fail at some point during development. Such investment is obviously at risk, subject to success at various preclinical and clinical development stages. In addition, potential manufacturing or safety concerns can also have a major financial impact in other ways:

(i) extend already long development timelines (reducing market exclusivity period);
(ii) require additional investment in process development, repeated work, or implementation of corrective measures;
(iii) prevent a programme from entering or progressing towards later stages of clinical development;
(iv) cause the failure of a programme during clinical trials, requiring a repeat of the trials or stop or delay final commercial approval due to quality or safety concerns;
(v) require considerable investment in process redesign and adaptation, reformulation, or even resolving product recalls.

These issues can be worth many millions in lost opportunities or investments lacking a return. In this context, it is desirable to select or design a successful candidate early on by asking the right questions.

## 2. Developability as an Intrinsic Part of QbD

The original definition of quality by design (QbD) by Juran and others [6, 7] established the importance of understanding customers' needs (customers defined very broadly) and designing product features and performance to satisfy those needs, as well as processes able to produce those features, not only in terms of manufacture, but also storage and distribution, utilisation, reimbursement, and so forth. As recognised by several authors, the implementation of QbD methodologies to the development of new therapeutics requires the definition of a quality target product profile (QTPP) as a basis for performance, and the identification of those quality attributes that are critical (CQA) and need to be controlled carefully to maintain product integrity and efficacy [8]. However, current QbD implementation, as defined by current ICH Q8(R2) and subsequent guidelines [9] (http://www.ich.org/) is primarily limited to manufacturing process understanding, but does not integrate product knowledge aspects, such as product design and product specifications for intended use.

In this context, “developability” can, in fact, be considered as an extension of QbD guidance, providing a bridge between “product knowledge” and “process understanding,” addressing the influence of product characteristics in manufacturing and clinical outcome, and helping expand the design space for a drug candidate. We show how developability can be applied to early derisking and how it can be seamlessly integrated with both discovery and process development activities.

Any new therapeutic candidate needs to answer the following questions: can it be made (at the right cost)? Is it stable? Can it be formulated for the intended route of administration? Is it safe for patients? Can it access the target tissue/organ at the required dose and during an adequate time window? Will it produce the intended biological activity and show sufficient effectiveness in patients?

Even before a lead candidate is found, such requirements can be summarised in an intended performance profile, and from that profile one can derive the required characteristics that will help ensure the development of a high quality therapeutic candidate. In this context, developability addresses more than simply “purity” or “stability” aspects of the manufactured product. It also provides a platform to incorporate early on a solid basis for “product knowledge” and defines, right from the outset, a robust QTPP that would greatly increase the odds of a successful, safe, and efficacious drug product (see ICH Q8(R2) for a definition of QTPP).

**Figure 1:**
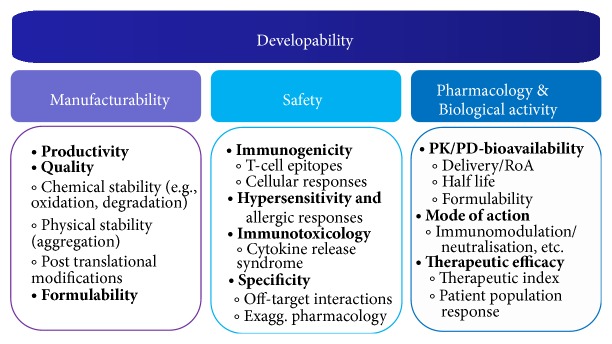
Developability rests in three main “Quality areas” or “pillars”: Manufacturability, Safety-Toxicity, and Pharmacology & Biological Activity. Abbreviations: RoA: route of administration; PK/PD: pharmacokinetics/pharmacodynamics. Adapted from [5, 10].

The subject of developability has been covered quite thoroughly in other publications [5, 10]. In short, the developability profile of a given new drug candidate is sustained by three “quality areas” or “pillars” that ultimately define its performance (Figure 1).

(i) *Manufacturability*: the main purpose of this type of assessment is to evaluate whether a given product can be manufactured with the expected quality characteristics, stability, and purity, at an assumable cost and able to be formulated for the intended route of administration.
(ii) *Safety*: biologics usually lack the toxic effects seen in small molecules, for example, associated to their metabolism, and so forth. However, immunogenic and hypersensitive reactions are a growing area of concern, as we will see later. Also, events associated with lack of specificity (off-target) or “exaggerated pharmacology” (on-target) can potentially compromise the therapeutic window for a particular product.
(iii) *Pharmacology & Biological Activity*: the third pillar consists of longstanding critical issues, which are becoming an important aspect for many biologics. For example, half-life, compatibility with specific formulations (i.e., sustained release) and routes of administration and “effective” concentration at target tissue are very important aspects that can influence the efficacy of a treatment. Also, early assessment of mode of action (particularly in immunomodulatory products) and patient segmentation and dosing can provide useful information that could potentially help designing clinical trials and increase likelihood of success.

These categories are also interrelated. Low stability can cause aggregation and thus safety issues (immunogenicity). Also, the ability of a product to be formulated for a specific route of administration can impact the bioavailability and pharmacology (and hence the efficacy) of a given candidate. Along with these “quality pillars,” the QTPP will ultimately define what the requirements for a given product are, so it can be considered to be “fit-for-purpose” aligning with each of the areas just defined:

(i) *Fit for process*. It can be manufactured at the required scale using standard processes. It is sufficiently robust to endure process excursions without impacting significantly CQAs. It is stable enough to endure process and formulation requirements (Manufacturability).
(ii) *Fit for patient*. It achieves desired therapeutic outcome without compromising patient safety. Does not introduce potentially dangerous side-effects (Safety).
(iii) *Fit for indication*. It is suitable for required disease condition, dosing regime, patient population, route of administration, and circulating half-life (Mode of Action & Pharmacology).

## 3. Beginning with the End in Mind

### 3.1. Defining a QbD Workflow

**Figure 2:**
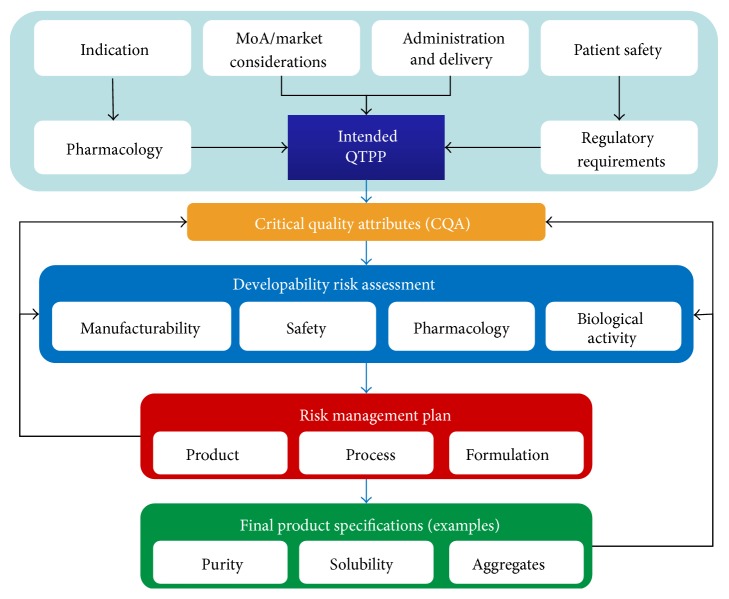
Developability flowchart. The flowchart illustrates how a development plan could be articulated to integrate effectively developability risk assessment tools. Setting an intended performance profile (QTPP) based on indication, pharmacology, mode of action, market, and delivery, amongst other considerations, allows the developer to determine the CQAs against which the lead candidate and process should be evaluated. A developability risk assessment would help identify specific risks impacting those CQAs and design and implement a risk-mitigation plan. This might involve modification in the selection or design of a lead candidate, potential reengineering (product), designing specific elements of the manufacturing process aimed to minimise or control risk, or perhaps some specific formulation requirements. All these steps will define the final product specifications in terms of measurable and controllable characteristics.

The implementation of a developability risk assessment requires a good understanding of the intended product properties and performance.  Figure 2 provides an illustration of how an ideal development workflow could be structured and how developability risk assessment sits at its core. One important aspect to notice is that, for this workflow to operate successfully, an adequate Quality Target Product Profile (QTPP) needs to be defined right at the outset of the drug development process (See ICH Q8(R2) for a definition of QTPP). This should be done during early discovery stages (product design) in order to formulate in the highest possible detail the intended performance, safety, and economic target profile, which will ultimately determine the product characteristics to aim for during the drug development process.

From this starting point, CQAs can be derived and a suitable developability risk assessment implemented to either derive optimal candidates matching the required CQAs or redesigning lead candidates that are able to match the target profile. Indeed, during the design stage these attributes should be mapped out and introduced or selected in the lead candidates but, of course, they should also be part of the design and optimization of the manufacturing process, so that such attributes can be properly controlled in an effective way. One might expect that the derisking methodologies introduced early on during candidate design and selection will, in turn, increase the robustness of the manufacturing processes, making it easier to control specific CQAs and minimising the incidence of deviations or out-of-specification (OOS) excursions.

The definition of a relevant QTPP is not a simple task. It does require the involvement of technical experts from multiple disciplines and areas of development (discovery, manufacturing, and clinical development), supply chain, distribution, and so forth. Most importantly, it should also incorporate key input needs and requirements from end-users or what in QbD nomenclature is known as the “voice of the customer.” It is important to note that “end-users” or stakeholders should be defined in a broad sense to ensure success (in concordance with the definition of “Big Q” by Juran) [7] and should include patients, clinicians, payers, and health care provision agencies, as well as input from discovery, manufacturing, regulatory, supply chain and commercial functions.

QTPP characteristics will relate to the desired indication, patient population, drug target, and dosing regime; the route and method of delivery; the target indication and market; the manufacturing platform; the specific molecular format to be used; and the inherent properties of the product. It is important that the QTPP arises from consideration of the whole life cycle of the drug, from design and manufacturing to distribution and patient administration and, even very importantly, its potential utilisation in additional indications in the future that could incorporate very different specific requirements for the product.

### 3.2. Developability: A Three-Stage Process

A developability assessment programme basically consists of three different stages.

#### 3.2.1. Risk Assessment

The simplest and most cost-effective way of assessing risk is by implementing computational approaches able to predict specific developability features by using the sequence of the biopharmaceutical candidates as a single input. These methodologies can have an extraordinarily high throughput and are relatively simple to implement. As we will see later on, suitable proxy analytics (high throughput) can also be utilised for this type of assessment.

#### 3.2.2. Implementation of a Risk-Mitigation Strategy

Depending on where in the process the risk assessment has been performed, different courses of action can be considered. In the case where process development (i.e., cell-line development) has not been initiated, two different routes can be explored: (a) selecting alternative candidates with a better risk profile and (b) redesigning a candidate to correct issues highlighted by the risk assessment [11]. If, by contrast, the product has already been taken into process development or in cases where reengineering is not an option, process-related interventions can help mitigate some of these potential problems. These could potentially include the screening larger numbers of clones during cell-line development or the utilisation of alternative downstream processes amongst others.

#### 3.2.3. Validation of Course of Action

The developability risk-mitigation cycle is completed by introducing appropriate validation studies. For example, in the case of immunogenicity of biopharmaceuticals, candidates can be reengineered to eliminate the occurrence of specific T-cell epitopes in the sequence and then tested using relevant cell-based assays that make use of blood samples from human donors.

### 3.3. Developability Methodologies

There are a number of different methodologies that can be used to assess different developability aspects relevant to biopharmaceutical products [5]. Two main approaches involve the use of computational methodologies alongside suitable* in vitro* assays.

#### 3.3.1. Computational Tools

The use of computational tools in early development is experiencing a growing attention due to their relative simplicity of implementation and flexibility, providing considerable benefits in terms of high throughput, low cost, and relatively short time of analysis. They can also be applied at any given point in time, given that they are usually not limited by material availability or assay constraints. These methodologies make it possible to begin building product understanding as soon as the sequence of a candidate is known. They offer a window onto properties that would otherwise not be available until much later in the manufacturing or clinical development process, and can help build quality into the product by selecting or designing lead candidates with favourable characteristics. Currently, there are a number of computational methods available for the prediction of immunogenicity (Safety) and physical and chemical stability (Manufacturability) of biopharmaceuticals, amongst other properties [5]. And we expect that in the near future, computational methods will also be able to assist in the design of purification protocols or formulation compatibility [12, 13].

#### 3.3.2. Surrogate/Proxy Analytical Tools

Standard process analytics are often not “fit for purpose” in an early developability assessment context, primarily because of limitations in throughput, assay time, resource, or material requirements. Therefore, there is a drive towards methodologies that could potentially reduce material requirements by as much as 10^3^-10^4^ fold as well as increasing sample throughput by 10^2^-10^3^ fold. Obviously, these methods cannot provide the same level of information than that is achieved by standard analytical technologies. In many cases this will mean that a “surrogate” or “proxy” assay is sufficient to assess a given property for a product candidate. The analytical methods used in early-stage development are undergoing a rapid development towards miniaturisation and high-throughput analysis [11, 14–21] and their integration with early, rapid, and low-cost analytical and computational methods lie at the heart of the concept of “Developability.” We have reviewed examples of such methodologies elsewhere [5].

### 3.4. Key Areas for Developability Risk Assessment

#### 3.4.1. Protein Aggregation and Chemical Stability

Aggregation and degradation are two particularly important issues that can appear at various stages of biopharmaceutical development. They can affect negatively the yield and economics of the manufacturing process but also can impact the performance of the product and, ultimately, patient safety [22, 23]. From a manufacturing perspective, tackling aggregation and chemical degradation through process design can be complex and costly. In the clinic, the presence of aggregates in biopharmaceutical preparations can be harmful to patients [24], and also can increase immunogenic reactions in patients [25, 26]. Formulation and container-closure interactions with product can also enhance aggregation, with potentially devastating effects in patients [27–29]. Furthermore, besides aggregation, the incidence of chemical degradation or posttranslational modifications (PTM) can also have a negative impact on the immunogenicity and safety of biological therapeutics [30]. For example, some specific PTMs, such as abnormal (non-human) glycosylation, can increase the incidence of anaphylactic reactions to biopharmaceuticals [31].

Over the years, a number of different models have been developed to predict the intrinsic aggregation propensity of proteins, and many of them have been reviewed elsewhere [5, 32–35]. Aggregation prediction algorithms are generally useful when comparing the aggregation propensity of highly similar candidates (i.e., sequence variants of a parental molecule) and also for detecting and disrupting aggregation hot-spots through protein-engineering methods. However, it is still challenging to assess the aggregation risk of a given biotherapeutic in the absence of a reference protein of similar nature for which experimental aggregation properties are known.

We have recently developed an antibody-specific algorithm to predict aggregation, based on experimental data obtained by expressing several hundred of antibodies in a CHO-GS mammalian expression system and further validated in a collection of 50 unrelated antibodies with good predictability results [36]. This tool can be used to assign molecules to two different classes (*Low* and* High* aggregation risk), using sequence and structural descriptors as input. This classification uses a pre-defined cut-off calibrated experimentally as an indicator of relative process risks linked to aggregation events during process development and manufacturing. Methods such as this are an important step in implementing high-throughput and inexpensive aggregation assessments that can be incorporated into a simple and actionable manufacturability risk.

Modifications in the chemical composition of biopharmaceutical products, whether due to cellular processes, enzymatic or chemical and degradation reactions, can result in a complex level of product microheterogeneity. It is estimated that up to 10^8^ different species could be found in a single vial of a biopharmaceutical product [9]. An in-depth review of different types of chemical instabilities and PTMs can be found elsewhere [37, 38]. These include degradation pathways such as deamidation, oxidation, and isomerisation, as well as undesired glycosylation.

Many of these modifications are sequence-specific and can be predicted using computational approaches. However, generally speaking, not all the instances of chemical degradation or PTMs are equally relevant to the performance of a given biopharmaceutical product. For example, their proximity to the active site of the molecule or potential role in significant product degradation might increase their potential risk. For example, Asparagine deamidation and Aspartate isomerisation, two of the most commonly found chemical instabilities in antibodies, can either have very little impact on stability and functionality of the molecule or, in severe cases, can potentially cause loss of activity, high product heterogeneity, and promote aggregation and fragmentation. The incidence of these modifications is influenced by pH, temperature, sequence, and solvent accessibility [39]. These types of instabilities can potentially be managed by process control and formulation [40–42], but they may also, in some instances, require protein engineering due to their high impact on product quality [43].

Product heterogeneity and instability can also be the result of cellular processes such as glycosylation. Proper glycosylation is important not only to confer specific biological characteristics to a given biopharmaceutical, including its potency and pharmacological properties [44, 45], but it also can be a determinant factor in the adequate folding and assembly of a product. It also often defines other key attributes, such as stability, solubility, and immunogenicity [31, 46]. Undesired glycosylation can, in occasions, interfere with the biological activity of a biopharmaceutical. Furthermore, it is also important to mention that the presence of nonhuman glycans in a product is a known risk for hypersensitivity and anaphylactic reactions to biopharmaceutical products [47]. In addition, finally, chemical glycation during bioprocessing, due to reaction with sugars present in culture media, can potentially introduce product heterogeneity resulting in aggregation, stability and potentially immunogenicity issues. Susceptible sites for glycation can be predicted. However, forced glycation studies could be more useful in confirming not only susceptible positions, but also in helping to define the magnitude of the problem as well as determine the conditions that promote or prevent its occurrence.

#### 3.4.2. Productivity and Yield

There is one important aspect not often recognised in biomanufacturing, and it is the relationship between productivity and product stability (primarily aggregation). As we have described before [48], protein aggregation and stability do, in fact, have many different “faces.” Aggregation can also appear in the form of intracellular inclusions, low cell/culture viability, or low levels of productivity. This is primarily due to the fact that biological systems have developed an array of tools and systems specially tailored to prevent misfolding and aggregation. However, industrial requirements are often not properly matched to the capabilities of the biological platforms used in biomanufacturing. For example, in the case of mammalian cell hosts, upon the occurrence of a misfolding event, proteins are held in the endoplasmic reticulum and either pushed towards a refolding or a degradation pathway. Therefore, unstable products would naturally have a lower chance to be secreted. We have observed such behaviour particularly in mammalian systems, linking high-aggregation propensity with low productivity. Clonal selection can, occasionally, offset the intrinsic challenges contained within the polypeptide chain to be expressed. However, we typically observe a high degree of correlation between productivity and aggregation.

**Figure 3:**
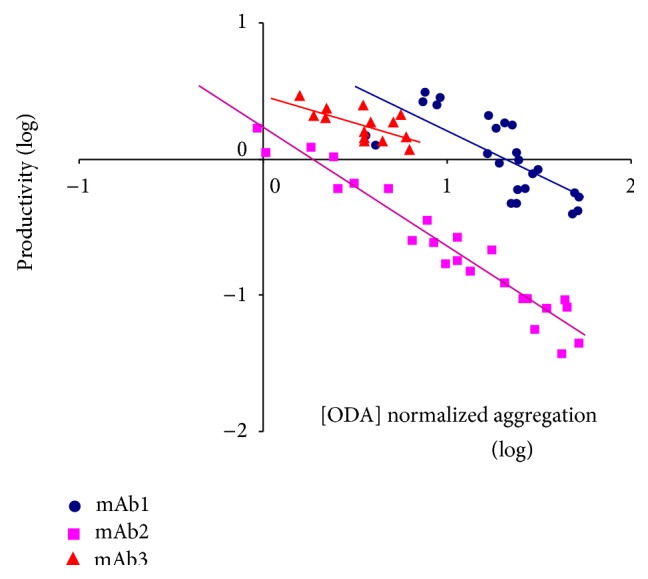
Correlation between antibody productivity and aggregation in three different antibody families: mAb1, mAb2, and mAb3. Variants were derived from three different parental antibody molecules by incorporating single and double sequence modifications. All different antibodies were expressed transiently under identical conditions to minimise any clonal variability in expression. Relative aggregation was assessed using Lonza's Oligomer Detection Assay, or ODA [11].

Figure 3 shows one example of such correlation in three different antibody families that were built from three different parental monoclonal antibodies by incorporating single and double mutations. Similar patterns have also been described by our group in instances where biopharmaceuticals had been reengineered to reduce their aggregation levels. We therefore believe that aggregation prediction could be potentially utilised as a surrogate for productivity levels, particularly in biopharmaceuticals expressed in mammalian systems. Furthermore, we and others have found a correlation between the amino-acid composition of specific areas of the antibody molecule and the productivity observed in mammalian systems. These observations open the door to the development of predictive platforms that could be used to assess product expression by means of computational tools [49, 50].

#### 3.4.3. The Importance of Formulation: Formulability Assessment

Formulation and its impact in the delivery of biopharmaceuticals are gaining increased attention in the industry. Formulation can influence the pharmacology of the product and its efficacy, as discussed earlier, but also can have an important impact on other vital product attributes that are linked to patient compliance and even costs associated with a given treatment. For example, in some extreme cases, the costs associated with the infusion of a biopharmaceutical product, in a hospital and under specialised supervision, could surpass the cost of the product dose itself [51]. Therefore, there is a growing interest in formulations and delivery methods that could facilitate self-administration as well as increase patient compliance and reduce the total cost of treatment [52, 53]. Subcutaneous delivery presents a number of advantages compared to traditional infusion approaches. It is simpler, less invasive, reduces patients' discomfort, and can modulate the product pharmacology by facilitating a gradual/sustained release of the product. However, the delivery of a sufficient dose typically requires high product concentrations (100–200 mg/mL for a monoclonal antibody). The use of such high-concentration formulations introduces new challenges in the form of solubility constraints, high viscosity, aggregates, and phase separation that could make subcutaneous delivery unsuitable for a given product [21, 54].

Therefore, a good understanding of the suitability of a given product for a required formulation and RoA (“formulability”) can be crucial very early on in product development. Moreover, “formulability” is also very important in other areas of development. For example, product losses are unfortunately common in cases where products are not stable in a given buffer or do not tolerate a specific pH range. This is also the case where products need to be concentrated during the manufacturing process. Drug-substance storage during downstream processing can require concentrations ranging from 25–200 g/L, because of limitations in volumes that a plant can store at a given time. This problem has been exacerbated by the increase in product titre that can be achieved in today's manufacturing platforms [55].

Formulability assessment is still a nascent area and we still lack simple platforms to assess the suitability of a given product candidate to be formulated at high concentrations (i.e., for subcutaneous administration) or compatibility with basic solution and process conditions. Formulation screening can be informed by computational methods in terms of aggregation propensity, long-term stability [56], and selection of excipients [57]. However, a number of high-throughput strategies have been proposed to assess protein stability at high concentrations as well as viscosity [15, 18–20, 58]. Furthermore, the use of computational methods can help make the formulation screening process more manageable. One interesting approach involves combination of machine-learning computational tools with high-throughput analytical tools, allowing the design of biopharmaceutical formulations with very limited product availability and early on in the development process [14]. Furthermore, computational methods are also useful in integrating measurements from different orthogonal analytical methods, potentially allowing the analysis of large data sets. Examples of this type of approach include Chernoff faces, star charts, and Empirical Phase Diagrams [59].

#### 3.4.4. Safety in Biopharmaceuticals: Immunogenicity and Immunotoxicology

Biopharmaceuticals are generally considered to be relatively safe to patients when compared to small molecule therapeutics. However, their administration to patients can cause a number of undesirable side effects, usually related to pharmacology issues, mechanism of action or, more commonly, immunogenic reactions [60, 61]. Immunogenicity is often considered to be one of the principal safety concerns for biotherapeutics and one of the primary causes for attrition during early clinical development.

Current clinical data suggests that the majority of therapeutic proteins are to a variable extent immunogenic [62]. The generation of an unwanted immune response can negatively influence both the efficacy and safety of the therapeutic protein. Therefore, the incorporation of an immunogenicity assessment early on during preclinical drug development can significantly reduce the risk of generating an unwanted immune response in the clinic, which could potentially modify the pharmacology of the product or render it completely inefficacious. In extreme circumstances, biopharmaceuticals can also cause severe hypersensitivity, anaphylactic or immunotoxicology reactions that can put a patient's life at risk [31, 63–65].

In general, immune responses to therapeutic proteins are assessed in the clinic by monitoring the generation of antibodies raised against the protein. However, regulatory bodies encourage innovators to explore the use of preclinical methodologies that could give an early indication of immunogenicity risks to patients, including both* in silico* and* in vitro* methodologies [66–68].

Immunogenic responses to biopharmaceuticals (humoral or not cell-mediated) can be either T cell dependent or independent. T cell independent antibody responses are generated when B cells are able to recognise and bind to epitopes in the protein, but in the absence of T cell help these are generally low affinity, transient IgM antibodies. When a T cell response is also induced by the therapeutic protein then the antibody response can lead to high-affinity, long-lived IgG antibodies, which are much more likely to affect the safety and efficacy of the therapeutic protein in the clinic. Due to the importance of the T cell response in the development of long-lived, high-affinity antibodies, there is much focus on the identification and removal of T cell epitopes during the development of therapeutic proteins to reduce their potential immunogenicity risk.

During the last two decades a number of computational methodologies have been developed for the prediction of immunogenicity. Most of these tools assess the T cell epitope content in proteins by predicting the binding specificities of peptide fragments from the protein of interest to HLA class II receptors. Such tools are reviewed elsewhere [69, 70].* In silico* T cell epitope profiling tools can be efficiently applied during the lead selection and optimisation stages in three ways: (a) to rank protein leads based on their relative immunogenicity risk, (b) to identify specific peptides within a protein sequence with high immunogenicity risk, and (c) to guide protein reengineering by helping remove T cell epitopes, a process known as deimmunisation. The efficacy of many of these computational approaches has been validated in the lab using, amongst others, HLA binding assays or* ex-vivo* T-cell activation assays that we describe below. However, one common question often asked is whether such computational platforms are effective at predicting immunogenicity in a clinical setting. On one hand, most of such tools use HLA binding as the “main trigger” for immunogenic reactions; however, as we discuss in this paper, immune responses involve multiple cellular and humoral components and are subject to the influence of many different elements that would be impossible to encode in an algorithm in a simple way, including genetic and disease-related patient variability. On the other, validation of the efficacy of such algorithms would require testing many different protein molecules with controlled variations in sequence (and potential T-cell epitopes), standardised formulations, route of administration, aggregation content, and so forth in a sufficiently large number of patients providing a good coverage of different HLA halotypes and controlling any potential disease-related influence. Besides being ethically inadmissible by any regulatory agency, such trials would be extremely expensive for any standards. However this does not mean that some degree of validation is not achievable. For example there are studies confirming the clinical safety of biopharmaceuticals that were previously assessed using such computational methods [71, 72].

*In vitro* and* ex-vivo* cell-based assays have the advantage of being able to evaluate and characterize the immune response to a therapeutic protein in a fully human system, thus providing important information on the safety of the protein prior to first-in-man trials. Human* ex-vivo* cell-based assay platforms have the additional advantage of being able to assess much more than just the potential T cell epitope content of the primary amino-acid sequence. These assays can also include the analysis of any conformational epitopes (e.g., B cell epitopes), impurities (e.g., aggregates or particles), and contaminants (e.g., host cell protein, endotoxin) present in the protein sample. A number of fully human* ex-vivo* assay platforms, including T-cell activation assays, and so forth are currently being used to assess immunogenicity risk, and have been reviewed in more detail elsewhere [5, 70].

In all these assays, the source and quality of the human primary cells used for the* ex-vivo* assays are of critical importance. Donors should be selected to match the intended target population (e.g., a global population that would closely represent a Phase I clinical trial). Moreover, blood samples can be sourced from patients suffering from a specific disease indication or with a given genetic or ethnic background that could be relevant for the therapeutic agent being developed. For example, PBMCs can be sourced from patients suffering from rheumatoid arthritis to assess their response to a therapeutic protein being developed to treat this condition, thus taking into account both the immune status and genetic background (i.e., HLA allotype makeup) of the intended patient population. The use of PBMCs taken from patients with the targeted disease indication may ultimately be more representative of the type of immune responses that could be observed in subsequent clinical trials.

T cell assays are frequently used as a key indicator of the potential immunogenicity of a given product. T cell activation can be assessed by means of intracellular cytokine expression or cytokine secretion as well as cell surface activation marker and proliferation [73, 74]. In the case of T cell assays, the format of assay is very important, and a number of product-related factors should be considered when selecting the most suitable approach. These include the nature of the protein (e.g., peptides, antibodies, antibody fragments, novel protein scaffolds, fusion proteins, and recombinant proteins), mode of action of the protein (e.g., toxic or immunomodulatory proteins can interfere with some assays), and the purity of the protein (e.g., some assay formats are more sensitive to endotoxin and aggregates). Often an optimisation of the intended assay format is required to ensure that the most suitable assay format is being used for the therapeutic protein. Optimisation parameters often include protein dose, kinetics of the assay, and interference in the assay (e.g., coculture with a positive control to identify any inhibitory effects of the test protein).

There is increasing concern about the prevalence of preexisting antibodies to many of the novel protein therapeutics that are currently being developed. Many novel protein scaffolds and small antibody fragments are being modified to extend the half-life of the molecules. One such half-life extension technology is PEGylation, and there are recent reports showing that up to 20% of the healthy general population has detectable pre-existing antibodies to PEG [75, 76]. Some novel antibody scaffolds have also reported problems with preexisting antibodies in the clinic [77], leading to significant delays and increased costs associated with identifying B cell epitopes and reengineering the molecule. The prevalence of preexisting B cell responses against a given therapeutic protein can be assessed in PBMC samples from human donors to determine the production of antibodies that could cross-react with the therapeutic protein being assessed [78].

#### 3.4.5. Aggregation and Immunogenicity

There is a well-documented link between the incidence of aggregation in biopharmaceuticals and observed immunogenicity in the clinic [26]. However, the majority of therapeutic proteins contain at least a low level of aggregates, and it is not currently known what type and amount of aggregation can pose a risk for increased immunogenicity [79]. Some examples of the relevance of aggregation in immunogenicity include erythropoietin or interferon. Eprex is a human erythropoietin (EPO) which underwent a formulation change that was subsequently linked to increased antibody formation to the endogenous form of EPO. This increased immunogenicity was associated with the development of pure red cell aplasia (PRCA) in patients treated with this product. There have been multiple explanations of the reasons behind this immune response, but one of the most prevalent views seems to associate the incidence of PRCA with the increase of product aggregates upon changes in formulation and enclosure systems utilised in the manufacture of the product [28, 29]. Another example is IFN*β*1a, prescribed for the treatment of Multiple Sclerosis (MS) in the clinic. Out of the two products currently registered for clinical use, Avonex and Rebif, the former seems to induce low levels of immunogenicity in patients (approximately in 2% of patients), whereas Rebif seems to be highly immunogenic (with approximately 25% of patients developing antibodies against the drug) in MS patients. In this particular case, the observed rates of immunogenicity can be linked to levels of aggregates found in each of the products, with Rebif exhibiting higher levels of aggregation than Avonex [80]. There is also recent data suggesting that the aggregation of monoclonal antibodies can lead to a significant change in the presentation of potential T cell epitopes by dendritic cells* in vitro* [81].

#### 3.4.6. Preclinical Immunotoxicology and Hypersensitivity

A large proportion of therapeutic proteins both in commercial use and in development have a mechanism of action reliant, at least in part, on immunomodulatory activities. This also raises the risk of overstimulating the immune system and potentially increasing the chances of an immunotoxic response to the therapeutic protein. This was clearly seen during first-in-man trials for the anti-CD28 agonistic monoclonal antibody TGN1412, where a severe inflammatory response was induced in treated patients. This response included cytokine release syndrome (CRS, or “cytokine storm”) and multiple organ failure. Subsequent studies have indicated that it was the CD28 agonistic activity rather than any sample contamination or errors in the manufacturing, formulation, dilution or administration of TGN1412 that led to the CRS response [63, 82–86]. In this particular case, preclinical studies both* in vitro* and* in vivo* failed to predict the induction of CRS, mainly due to suboptimal conditions using human PBMC* in vitro* and differences in the immune system between humans and primates* in vivo* [87]. More recently, a number of new* in vitro/ex-vivo* assays are currently being developed that seem to be able to detect CRS responses and, therefore, have been proposed as a new tool to assess this type of risk during preclinical development of therapeutic proteins [88].

Hypersensitivity and anaphylactic reactions to biopharmaceuticals can negatively affect patient safety and the development of new treatments. In some cases, such adverse reactions to some biopharmaceuticals could be linked to pre-existing antibodies (IgA, IgM, IgG, or IgE) that recognise nonhuman epitopes present in the product, such as nonhuman glycoepitopes. Interestingly, it has been proposed that at least some cases of hypersensitivity to biotherapeutics could be associated with the presence, before the start of the therapy, of IgE antibodies able to react with the product [47]. All this suggests that this type of assay could perhaps help avert hypersensitivity or anaphylactic reactions before entering the clinic.

## 4. Targeting QTPP: Developability Applications

Developability methodologies can indeed have an important beneficial potential if applied during early stages of discovery, ensuring that the right quality attributes are designed into the chosen candidates for development. As indicated above, this requires the combination of both predictive computational tools and adequate surrogate or proxy* in vitro* methodologies. As we have discussed, there are areas where new developments are needed to produce better predictive approaches and ultimately a balance needs to be achieved between both approaches to maximise outcomes. We have discussed elsewhere how such a balance could be articulated at different stages of development and, more importantly how different decision-making tools could be defined to achieve effective solutions that would improve success during preclinical and clinical development [89].

**Figure 4:**
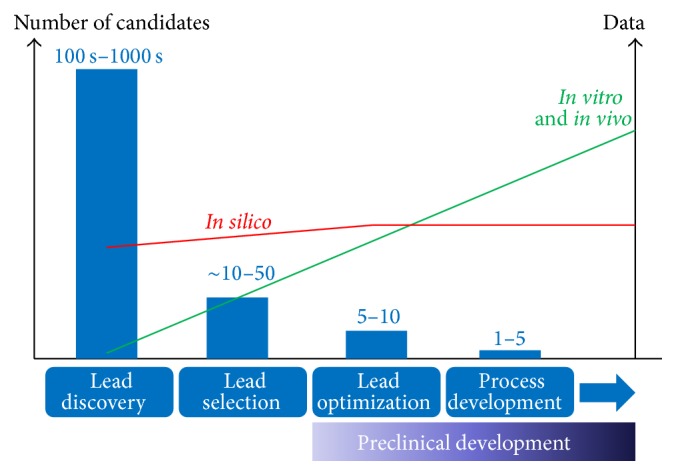
Implementation of developability methodologies in different stages of discovery and development and its relationship with number of lead candidates and available data. As the number of potential candidates converge into a smaller number, the amount of available experimental data increases.* In silico* computational methods can, by comparison, yield a lot of information at an early stage. As the product candidates progress in development, the introduction of* in vitro* analytics becomes feasible and an important element to help the decision-making process.

We would like to illustrate, however, that reliance on different approaches could evolve during the different stages of development for a new therapeutic product. For example, during early stages of development of a biopharmaceutical (typically an antibody), large numbers of candidates are evaluated (e.g., binding candidates out of display libraries) and consequently a lot of data is generated. At these stages, the most effective strategy for a developability risk assessment would largely rely on the use of computational tools to categorise or flag (ideally automatically) high-risk candidates to help guide the selection of more favourable molecules to be taken into later stages of development. For example, the aggregation prediction tools described earlier could be used in combination with the assessment of potential chemical instabilities and immunogenicity risk scores (using T cell epitope prediction). This approach would integrate early on both physical and chemical stability together with immunogenicity risks in a simple, fast and inexpensive manner. Furthermore, as fewer and fewer different candidates move into successive stages of development, novel assessment approaches (*in vitro* and* in vivo*) become feasible, and as a result the amount of data generated using such approaches increases steadily (Figure 4). In this way, as the project moves further into preclinical development, computational assessments gradually transition towards experimentally verified data to assist in the selection of lead candidates to move into later stages of development.

*Practical Case Studies.* Below we describe the application of some of the developability tools described in this article to the selection and engineering of biopharmaceutical candidates with enhanced properties. We have chosen two different case studies to illustrate their implementation in different areas of risk. In both cases we comment on the application of the respective risk assessment, risk mitigation and validation steps and how both* in silico* and suitable surrogate or proxy* in vitro* methodologies can be combined in a unified workflow.

### 4.1. Engineering Antibodies with Improved Manufacturing Properties That Retain Biological Activity

The following case describes how a developability assessment and remediation programme can be utilised to ensure that manufacturing, safety, and efficacy requirements are included in the product specifications. As we indicated above, the definition of a relevant QTPP early on in the development of a new drug can be useful in identifying potential areas of risk and designing adequate (and inexpensive) remediation strategies.  Table 1 reflects some of the requirements for this particular product. One of the key criteria, often determinant in process development, is to achieve acceptable manufacturing costs and ensure patient safety by achieving a high product quality (linked to stability) and adequate efficacy, which will ultimately define to a great extent the performance of a product. As we have seen earlier, product instabilities in the form of aggregate, impurities or degradation are often responsible for the incidence of immunogenic responses in patients. These criteria therefore are often closely linked to specific CQAs, namely, aggregation levels (as well as other product impurities), product yields and, of course, biological activity. From these CQAs then a number of design criteria can be used to define a suitable developability programme. This is, however, not a trivial matter, given the fact that, often, stability problems in molecules such as antibodies colocate with biologically active regions of the molecule. This colocation is primarily due to the highly variable character of complementarity determining regions (CDRs) in the molecule. However, recent studies suggest that because of the nature of interactions involved, antibody-target binding regions are likely to be enriched in aggregation-prone regions [90].

For the purpose of this study we used a model monoclonal antibody with potential therapeutic applications. We selected a humanised anti-IFN*ɤ* antibody previously described in the literature [91]. In this particular case, the parental molecule (humanised antibody) exhibits significant aggregation problems both under native conditions (after capture step using protein A chromatography) as well as in accelerated stability studies (i.e., incubation at high temperature). Aggregate content was determined by gel-permeation HPLC methods (GP-HPLC), and, in some cases, monomer recovery (quantified by GP-HPLC) was used as a more precise way to assess protein loss due to aggregation and other factors.

With this case in mind, the design criteria chosen included the increase of product stability, reduction of aggregation, maintaining an adequate productivity and achieving all these requirements whilst maintaining acceptable affinity to target. The design plan, as described earlier, included a risk assessment, mapping areas of the molecule potentially responsible for the observed behaviour, and introduction of a mitigation plan that would involve the substitution of key residues in the molecule to improve the required parameters. Finally, the resulting product candidates would be assessed using relevant experimental techniques to determine whether the remediation plans satisfied the requirements for the product.

To this aim, three-dimensional structural homology models were built for the Fv regions of the humanised anti-IFN*ɤ* antibody. The molecule's sequence and structural properties were analysed using the latest version of Lonza's proprietary Aggresolve* in silico* platform to identify potential aggregation hotspots or “weak” regions that could justify the stability and aggregation issues observed in the molecule, as well as assessing the relative impact of specific amino acid modifications in the aggregation propensity of the molecule. Description of early aggregation-predicting algorithms and examples of their application to specific biopharmaceuticals can be found elsewhere [11, 92–95]. These analyses highlighted several potential aggregation hot-spots on the humanized antibody when compared to reference sets of monoclonal antibodies of known behaviour. After this analysis was completed, we selected a library of different sequence variants that targeted those potential aggregation hotspots as well as potential structural liabilities or “weak-points” that could influence the behaviour of the molecule. We further used structural information on the molecule and original murine antibody to refine this library and discard unsuitable modifications that could have a negative impact on the stability and structural integrity of the molecule or that could potentially impact its biological activity, for example, because of their physicochemical characteristics or proximity to key residues in the binding interface. After this secondary screening was completed a reduced number of variants were selected for further characterisation in relevant* in vitro* assays. (Homology three-dimensional models of variable domains of antibodies were built using standard commercial software (Accelrys's Discovery Studio—Biovia). Structural liabilities can be assessed by computing a variety of different parameters, such as structural alterations to key regions of the molecule (i.e., residues in close proximity to domain interfaces) or by assessing alterations in domain-domain interactions (i.e., energies of interaction or changes alterations in hydrogen bonds). In some cases, molecular dynamics simulations (Gromacs) can be utilised to assess changes in local flexibility that could affect the stability of the complex.)

Relative productivity of variants and parental molecules was assessed in suspension cultures of CHOK1SV cells, using small-scale transient transfections in 96 well plates. From these initial screenings two final variants were selected and expressed again transiently in suspension cultures using 200 mL flasks. These cultures generated sufficient material to perform confirmatory protein stability (aggregation) and activity studies. The main rational for using transient expression for this type of assessment resides in the fact that it eliminates any potential contribution of clonal selection in the observed product quality characteristics. Alternatively, pooled stable transfections can also be used successfully for this purpose.

**Figure 5:**
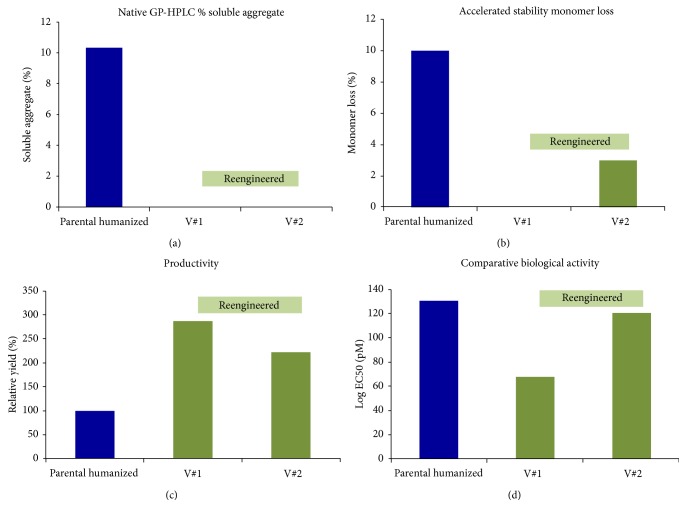
Reengineered antibodies display improved developability properties. Panel (a) shows the virtual absence of aggregation for both reengineered variants under native conditions when assessed by GP-HPLC. Panel (b) shows that the percentage of monomer loss after incubation 2 h at 60°C is significantly reduced in both reengineered variants, and virtually eliminated in V#1, indicating improved stability upon reengineering. Panel (c) shows that the productivity increases more than 2-fold in reengineered variants. Panel (d) shows that biological activity is not negatively impacted upon reengineering, with one of the variants V#1 showing increased affinity for the ligand.

After expression in culture, the two reengineered antibodies displayed significantly improved properties when compared to the parental humanised anti-IFN*ɤ*, validating the re-design approach taken. Specifically, GP-HPLC analysis showed an almost complete elimination of aggregation for the two selected candidates (Figure 5(a)). The same type of analysis after an accelerated stability study, in which the antibodies were incubated at 60°C for 2 hours, also showed positive results, with a significant reduction of monomer loss for both reengineered variants, compared to the parental molecule. Remarkably in one of the variants monomer loss was virtually undetectable after incubation at high temperature (Figure 5(b)). Furthermore, the observed yield of the reengineered variants increased up to three-fold when compared to the parental molecule (Figure 5(c)), in line with earlier observations in our group, linking antibody stability and aggregation to productivity. Also, very importantly, the reengineered variants also retained biological activity to similar or even better levels to those of the parental molecule (Figure 5(d)).

**Figure 6:**
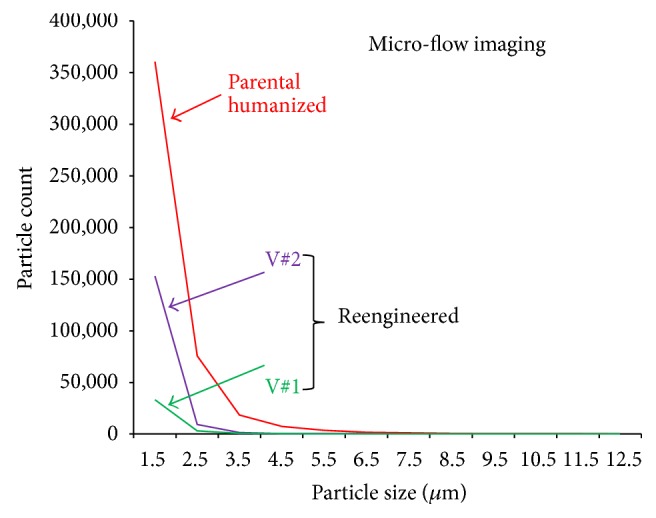
The amount of subvisible particles, including protein aggregates, in the humanized anti IFN*ɤ* and both reengineered variants was characterized using Micro-Flow Imaging (MFI). V#1 and V#2 variants display a 5- to 10-fold reduction in the number of particles below 3.5 *μ*m compared to the humanized anti IFN*ɤ*. Both reengineered variants contain no detectable particles over 3.5 *μ*m.

There is a growing concern about the presence of subvisible particles in biopharmaceutical preparations because of their potential impact on immunogenicity risk [96]. To address this all variants were analysed using Micro Flow Imaging (MFI). MFI is able to quantify a distribution of subvisible particles in a given protein solution based on their size. In our tests, the two reengineered antibody variants showed a significant reduction in particles across the spectrum when compared to the parental molecule (Figure 6).

These results, therefore, highlight how, the application of computational and adequate analytical tools during the initial stages of drug development can lead to a significant improvement in developability of a drug candidate. It also exemplifies the implementation of reengineering to control or improve essential design criteria that can have a significant impact in product quality attributes, thus decreasing the likelihood of quality and safety issues that could creep in during later stages of preclinical and clinical development.

### 4.2. Selecting Half-Life Extension Products with Reduced Risk of Immunogenicity Risk

This second case concerns with the design of strategies aimed to extend the half-life of biotherapeutic molecules, particularly small proteins, whilst controlling the potential incidence of potential immunogenic reactions in patients.

Why extended half-life and why immunogenicity? As we discussed earlier, the pharmacological properties of a biotherapeutic candidate can have a dramatic impact not only on the biological activity and performance of a product, but also can have a knock-on effect on healthcare costs (linked to administration of drug) and patient compliance. A product that requires daily administration is likely to face a substantial level of resistance by patients and payers, both in terms of costs and convenience. This can be exacerbated by the cost of producing the active molecule itself. For example, antibodies and other binding scaffolds usually need to be administered at relatively high concentrations in order to generate a suitable response in patients. As we have discussed earlier, this requires typically high product concentrations that also maintain relatively long circulating half-life in serum.

Antibodies are privileged molecules in this regard and they have been engineered by nature to remain circulating in the bloodstream for extended periods of time (typical half-life for an IgG is around 2-3 weeks). This is achieved through the interaction of the Fc portion of the antibody with the neonatal Fc receptor (FcRn) present in endothelial cells, which rescues antibodies destined for intracellular degradation, and reintroduce them in the bloodstream. Albumin also maintains long circulating half-life by a similar binding to the FcRn. As a result, a number of approaches have been proposed to extend the circulating half-life of biopharmaceuticals, including fusion or conjugation to Fc fragments, albumin, lipids (able to bind albumin) or albumin-binding proteins [97]. This is particularly important in the case of new “scaffold” molecules or alternatives to antibodies, which often are designed as small protein domains to increase their tissue penetration properties, particularly for the treatment of solid tumours.

Small molecules and proteins below the threshold for renal clearance (around 70 kDa) are rapidly cleared from circulation, in contrast to large proteins that have longer circulating half-lives. It is therefore no surprise that early strategies to extend the circulating half-life of therapeutic molecules involved the conjugation to polymers, such as polyethylene glycol (PEG), that are able to increase the apparent hydrodynamic radius of the product and hence reduce its clearance from the bloodstream through the kidney. However, recent developments are questioning the utilisation of PEG as an adequate approach to half-life extension. According to a long-held view, besides its impact in circulating half-life, PEG could also reduce aggregation of the product by hiding hydrophobic patches beneath a highly hydrated polymer shell, but also reduce immunogenicity by hiding potential T-cell epitopes present in the molecule [86]. Although this is indeed the case, a growing number of observations report an increase in the observed immunogenicity of biological drugs when linked to PEG. This is perhaps associated to the fact that many patients, in fact, possess pre-existing antibodies against PEG, likely due to earlier exposure to the agent from processed food, pharmaceuticals, and cosmetic products [75, 76]. Also there is the possibility that new epitopes could potentially be created upon conjugation of PEG to a protein of interest. Independently of its immunogenicity potential, the very high stability of PEG in the body is an issue. For example, there is growing suspicion of potential safety risks associated with the chronic administration of PEG, primarily linked to its accumulation in renal cells and the subsequent risk of renal failure [98].

It is therefore no surprise that alternatives to the use of PEG are being explored. In addition to increasing safety concerns referred above, the considerable increment in manufacturing costs associated with conjugated products are contributing to this interest. Many of these alternatives involve fusions to poly-amino acids that increase the apparent size of the molecule [99] or to moieties that take advantage of the recycling mechanism mediated by FcRn, described above.

With this background, the definition of a QTPP and associated CQAs and design criteria follow a similar approach to that described in the previous example, as reflected in Table 2. In this particular case, the main pharmacological drivers aiming to increase therapeutic index and reduce dosage can be achieved by extending the half-life of the product. As stated above, this can be done by increasing product size (passive mechanism) or by fusing the product to a “carrier” molecule that actively extends product half-life by using the “FcRn recycling” mechanism. In this particular case, the latter approach was utilised in the form of an albumin-binding domain. However, as we have seen above, it is important that the methodology employed to extend the product half-life also addresses safety concerns, primarily potential immunogenic reactions that could negatively impact the usability of the product. In this way it is important that methodologies aimed to reduce or eliminate T-cell epitopes are utilised as well as other potential contributors to immunogenicity (i.e., aggregation, degradation, or impurities).

In the case described here the chosen strategy for half-life extension of therapeutic proteins is to take advantage of the long circulatory half-life of human serum albumin (HSA) in plasma [100, 101]. The Albumod technology developed by Affibody is a proprietary albumin binding technology and is based on a small Albumin Binding Domain (ABD). This domain consists of a 5 kDa protein that has been engineered to bind HSA with high affinity and is designed to enhance the efficacy of biopharmaceuticals by extending their circulatory half-life in patients. The original ABD domain (ABD001, ABD3) was isolated from a bacterial protein, streptococcal protein G (SpG), which has the capacity to bind serum albumin. ABD001 had undergone affinity maturation, and one of the resulting engineered mutants, ABD035 demonstrated excellent stability along with an increased affinity for serum albumin of several species, including femtomolar affinity for human serum albumin [102]. ABD035 also retained an experimentally confirmed T cell epitope from ABD001 [103] and was therefore subjected to deimmunisation via protein engineering. A number of variants were designed to remove/reduce the number of T and B cell epitopes whilst maintaining thermal stability, solubility, expression yield, and affinity to HSA. The protein engineering stages were guided by B and T cell epitope prediction programs and available literature on ABD, and included iterative rounds of protein expression and analytical characterisation [104].

**Figure 7:**
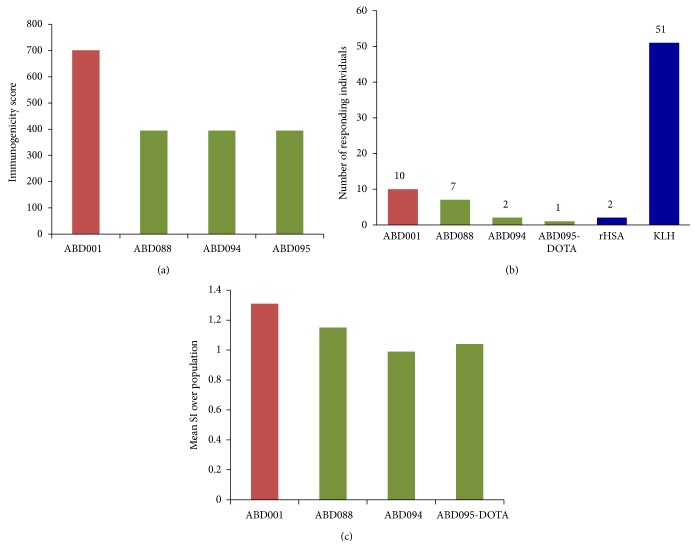
Immunogenicity assessment of ABD variants. (a) Predicted immunogenicity scores for three ABD variants and parental sequence ABD001. (b) Relative CD4+ T cell proliferation responses to ABD variants in a cohort of 52 donors, expressed as number of donors with proliferative responses to each of the ABD variants compared to negative (rHSA) and positive (KLH) controls. (c) CD4+ T cell proliferation responses to ABD variants in a cohort of 52 donors expressed as mean stimulation indices (SI) over the population. rHSA is used as a reference (SI = 1).

In order to mitigate potential immunogenicity risks we made use of the Epibase* in silico* immunogenicity prediction platform to select variants of the willd-type ABD001 with reduced immunogenicity potential. This platform has previously been used successfully in a number of biopharmaceuticals [105, 106]. The wild-type ABD001 and a total of 133 different engineered variants were subsequently screened for immunogenicity using the Epibase* in silico* platform, with profiling performed for the Caucasian population using 42 HLA class II allotypes. The variants were ranked based on their immunogenicity score incorporating DRB and DQ allotypes. Three variants, ABD088, ABD094, and ABD095, were then selected from the collection of variants based on their sequence, HSA affinity, thermal stability, solubility, and predicted lower immunogenicity risk.  Figure 7(a) shows the predicted immunogenicity scores for these variants and their comparison to the parental ABD001. Deimmunised variants were predicted to have a reduction of approximately 40% in their immunogenicity risk when compared to the parental molecule ABD001.

The wild-type ABD001 and 3 deimmunised variants, ABD088 and ABD094, and the conjugate ABD095-DOTA (DOTA—chelator for divalent metal ions) were further assessed* in vitro* for their ability to activate CD4+ T cells. During the* in vitro* immunogenicity assessment, proliferation of CD4+ T cells was used to monitor T cell activation response induced by the ABD variants. CD4+ T cell responses were assessed in PBMCs from 52 healthy donors representing the Caucasian population (frequencies based on HLA-DRB1 allotype distribution). Keyhole Limpet Hemocyanin (KLH) was used as a highly immunogenic benchmark protein and recombinant human albumin (rHSA) as a control reference. Data analysis included identifying the number of individual donors eliciting a significant CD4+ T cell response to each ABD variant and a measure of the mean CD4+ T cell response over the whole 52 donor population.  Figure 7(b) shows the number of donors with statistically significant proliferative responses using a blank control as reference. When compared to a blank control, only 2 out of 52 individuals responded to ABD094 and ABD095-DOTA, versus 10 donors responding to the wild-type ABD001. On the other hand, a total of 51 donors out of 52 responded to the KLH positive control and only 2 donors responded to rHSA.  Figure 7(c) shows the mean stimulation index (SI) over the population using rHSA as a reference for the four ABD variants (the Stimulation Index (SI) is calculated by dividing the number of proliferating CD3^+^CD4^+^ cells in the test condition by the number of proliferating CD3^+^CD4^+^ cells in the blank condition. The criteria for a statistically significant CD4^+^ T cell response was set at an SI value > 2 with an associated *P* value < 0.05. A cumulative count of the individual donor responses to each test protein over the 52 donor population was used to compare the test protein immunogenicity at a single donor level. The magnitude of the T cell response induced by each test protein was also calculated over the entire 52 donor population. To compare the population response to each test protein, the (geometric) mean SI value (with associated *P* value) was calculated compared to the reference (blank) condition and individual test proteins directly compared).

All three deimmunised variants show a reduction in T cell proliferation in comparison with the wild-type ABD001. The mean population response for ABD001 and ABD088 is statistically different (*P* < 0.05) from that for rHSA (mean SI of 1.31 and 1.15, resp.). The SI for ABD094 and ABD095-DOTA variants was not significantly different over the test population (mean SI of 0.99 and 1.04, resp.).

No significant* in vitro* CD4+ T cell response was detected against the lead candidate ABD094, indicating that the removal of T cell epitopes via engineering was successful in reducing the immunogenicity of the molecule. As a result of these studies, the candidate ABD094 was selected to progress into development and future clinical trials.

This project demonstrates the successful use of a combination of* in silico* predictions and* in vitro* immunogenicity assessment tools as suitable platforms to guide protein reengineering to remove T cell epitopes and to enable lead selection based on the relative immunogenicity risk of different candidates.

## 5. Conclusions

As drug attrition during development remains a critical hurdle, underpinning the scarcity of new therapeutic treatments that are both effective and affordable, a true, holistic implementation of QbD and Big Quality principles, as defined by Juran and Godfrey [7], is desperately needed in the industry. Existing ICH guidelines concerning the application of QbD to drug development provide mainly a structured framework for process understanding and characterisation. However, they do not emphasise adequately the relevance of product knowledge and design and their true impact on product quality as well as manufacturing and clinical outcomes. Despite of what it might seem obvious to most people, the industry still lacks the implementation of “true QbD” methodologies that start with the design of the product itself. We argue that the definition of a meaningful quality target product profile (QTPP) right at the inception of a new product, as well as the early determination of relevant CQAs and effective risk-management strategies, can facilitate this process. Indeed, having such clear sets of design requirements at the very beginning can help driving effectively the development of an appropriate manufacturing process with a higher probability of success.

Unfortunately, current standard practice in biopharmaceutical development usually makes use of highly fragmented and siloed processes. In our experience, this often means that many important product properties are not properly addressed during the design and lead selection stages and are left to be managed during manufacturing. This “traditional approach” can increase considerably risks for the product and can have negative consequences in product viability and development costs. We propose an alternative workflow that moves away from the classical linear-hierarchical development model into one that is more integrated and where adequate early risk-assessment tools can help controlling CQAs at a very early stage. This introduces a change in emphasis, by defining QTPP right at the outset and with a larger number of criteria that will ultimately determine the success of a given product (mode of action, target patient population, delivery requirements, etc.). Secondly, it does involve the introduction of additional derisking tools that increase the stringency of candidate selection, in order to meet the required QTPP, and that properly control CQAs in the product from the beginning of development. Ultimately we believe that such early risk-assessment paradigms can not only be financially beneficial in reducing development costs and the costs of “poor quality” (deviations, recalls, failed batches, or clinical inefficacy), but could also potentially accelerate the development of new product candidates, for example, by speeding up their transition from preclinical to clinical development.

The two examples chosen try to illustrate, in a very simple and succinct way, how this “early QbD” process could be articulated. We use a simplified and somewhat limited description of a target QTPP to show how CQAs can be subsequently derived and risk assessment and mitigation strategies can be rolled out. Furthermore, we describe how developability can in fact emerge as a “bridge” between discovery and development functions, raising CQAs awareness very early on as well as aligning the objectives of different stakeholders towards developing better, safer, and more cost-effective products. Indeed, developability assessment approaches are emerging as an important tool to expand on the current, still-limited implementation of QbD in the pharmaceutical industry, but as we describe, there are still many gaps that require further attention, such as early formulability assessment or more comprehensive safety profiling. We are also aware that, in our examples, many other aspects relevant to biopharmaceutical success have not been fully addressed, primarily due to limitations of space and scope of this manuscript.

As it happens with the introduction of QbD in commercial drug manufacturing, this “early QbD” approach still requires the definition of suitable tools to integrate information from different risk areas (relevant to CQAs) and “objective” approaches to decision making that are relevant to the intended performance of a given drug. For example, the evaluation of the relative importance of different risks during lead selection and their potential impact in product quality and clinical success remains a mayor challenge that needs to be tackled. We have started to address some of these issues elsewhere [89], but anticipate that alongside the introduction of predictive algorithms and surrogate analytics discussed here, the implementation of better data integration, and the development of objective decision-making tools will facilitate a more effective application of the methodologies reviewed in this article. This will, very likely, not only involve the development of new technical solutions, like computational models and tools, but also some degree of coordination and alignment across different industry stakeholders, from innovators to contractors to regulatory bodies to healthcare providers. We humbly hope that this review can help in fostering further discussions in the industry around this topic, ultimately biopharmaceutical quality and efficacy, which we believe will be essential for the long term success and sustainability of our industry.

## Figures and Tables

**Table 1:** QTPP, CQA, and design criteria derived for case study 1. In this particular case, previously known product shortcomings in terms of stability have been used to focus criteria around quality requirements for a hypothetical final product.

QTPP	CQA	Design criteria (Developability)
Optimise manufacturing and development costs Safety to patient	Aggregation Productivity	Increase product stability Reduce aggregation Maintain/increase product titre

Adequate product efficacy	Biological activity	Retain affinity to target within acceptable levels

**Table 2:** QTPP, CQA, and design criteria derived for case study 2. The main criteria considered in this particular example revolve around pharmacology and safety requirements for the final product.

QTPP	CQA	Design criteria (Developability)
Increase therapeutic index Reduce dosage	Extended half life	Increase product size (passive) Fuse/conjugate to “carrier”

Safety to patient Minimise resistance to drug	Low immunogenicity	Eliminate T cell epitopes Minimise aggregation
